# Elevated Lipoprotein(a) Predicts Recurrent Myocardial Infarction in Diabetic Patients Following Acute Coronary Syndromes

**DOI:** 10.1002/clc.70480

**Published:** 2026-10-31

**Authors:** Ermilo Echeverria‐Ortegon, Mario César Torres‐Chávez, Alfredo Pérez‐Tagle Tejeda, Paulina Caballero‐Osorno, Juan Pastor‐Cervantes, Hugo Laviada‐Molina

**Affiliations:** ^1^ School of Health Sciences Universidad Marista de Mérida Merida Yucatan Mexico; ^2^ Facultad de Medicina Mexicali Universidad Autonoma de Baja California Mexicali Baja California Mexico; ^3^ Memorial Healthcare System Hollywood Florida USA; ^4^ Department of Cardiovascular Disease Memorial Healthcare System Hollywood Florida USA

## Abstract

**Background:**

Patients with type 2 diabetes mellitus (T2DM) who experience acute coronary syndromes (ACS) remain at high risk for recurrent ischemic events despite secondary prevention. Lipoprotein(a) [Lp(a)] contributes to residual cardiovascular risk, but its prognostic impact on recurrent myocardial infarction (MI) and repeat revascularization in diabetic patients after ACS or percutaneous coronary intervention (PCI) remains unclear.

**Hypothesis:**

We hypothesized that elevated Lp(a) is associated with increased risk of recurrent MI and repeat coronary revascularization in patients with diabetes after ACS or PCI.

**Methods:**

We conducted a PRISMA‐guided systematic review and meta‐analysis. PubMed, EBSCOhost, and Cochrane Library were searched for studies evaluating Lp(a) and recurrent cardiovascular outcomes in patients with diabetes after ACS or PCI. Observational studies reporting hazard ratios (HRs) with 95% confidence intervals (CIs) were included. Pooled HRs were calculated using random‐effects models.

**Results:**

Seven studies comprising 7887 patients with diabetes and prior ACS or PCI were included. Elevated Lp(a) was associated with increased risk of recurrent MI (pooled HR 2.17, 95% CI 1.02–4.62; *I* ^2^ = 75%) but not repeat coronary revascularization (pooled HR 1.68, 95% CI 0.79–3.57; *I* ^2^ = 78%).

**Conclusions:**

Elevated Lp(a) was associated with increased risk of recurrent MI in patients with diabetes after ACS or PCI. Findings should be interpreted considering the observational study designs, substantial heterogeneity, and limited evidence for repeat revascularization. These findings support Lp(a) as a contributor to residual cardiovascular risk and its potential role in post‐ACS risk stratification.

**Trial Registration:** This study was prospectively registered in the International Prospective Register of Systematic Reviews (PROSPERO; registration number CRD420261296821).

## Graphical abstract

Elevated lipoprotein(a) levels are associated with an increased risk of recurrent myocardial infarction in diabetic patients following acute coronary syndromes or PCI, but not with repeat revascularization. These findings highlight Lp(a) as a key contributor to residual cardiovascular risk and a potential tool for post‐ACS risk stratification.

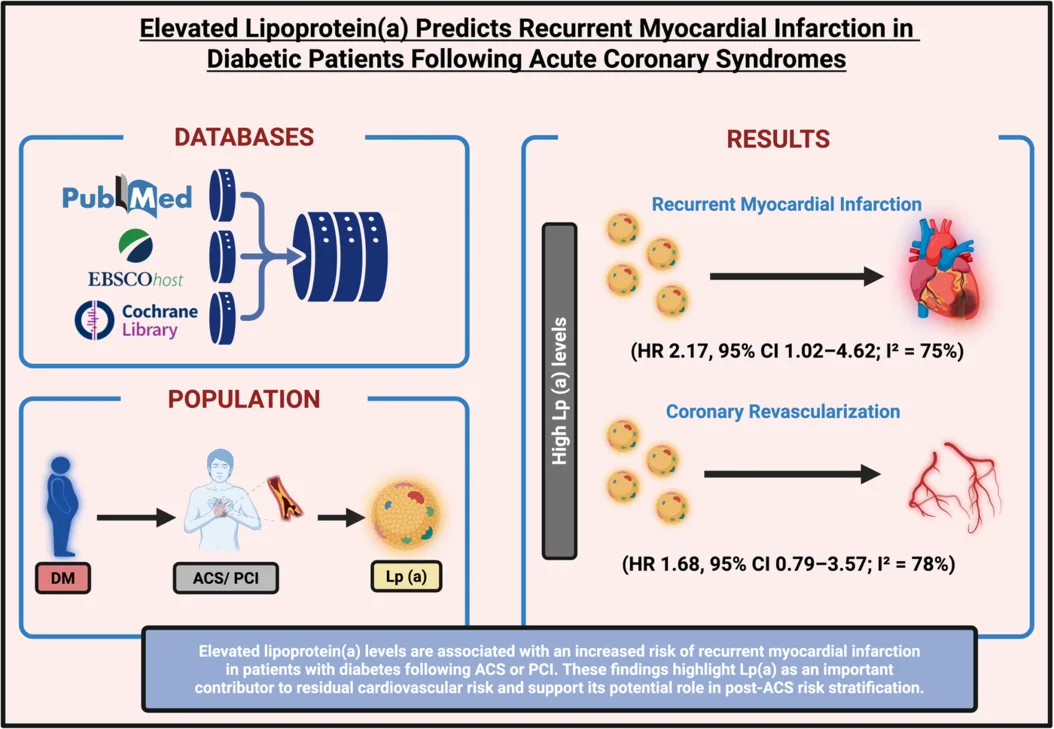

**Abbreviations**
ACS: acute coronary syndrome
DES: drug‐eluting stent
Lp(a): Lipoprotein(a)
MI: myocardial infarction
PCI: percutaneous coronary intervention
T2DM: Type 2 diabetes mellitus

## 1 Introduction

Atherosclerotic cardiovascular disease (ASCVD) remains the leading cause of morbidity and mortality among individuals with type 2 diabetes mellitus (T2DM), who experience a two‐ to fourfold higher risk of cardiovascular events compared with those without diabetes. Despite advances in interventional cardiology, patients with diabetes undergoing percutaneous coronary intervention (PCI) continue to experience worse clinical outcomes than their non‐diabetic counterparts, even in the era of contemporary drug‐eluting stents (DES) [1, 2].

Among patients with diabetes who survive a myocardial infarction (MI), the burden of residual ischemic risk is particularly pronounced. Prior studies have demonstrated that this population faces a substantially increased risk of recurrent ischemic events, which are closely linked to repeat coronary revascularization and cardiovascular mortality [3]. These observations underscore the need for improved risk stratification and prolonged post‐MI surveillance, as well as the identification of residual cardiovascular risk factors that may be amenable to targeted intervention.

Lipoprotein(a) [Lp(a)] represents one of the most prevalent inherited dyslipidemias worldwide, affecting approximately one in five individuals. Structurally, Lp(a) consists of an LDL‐like particle covalently bound to apolipoprotein(a), conferring both proatherogenic and prothrombotic properties [4]. Growing epidemiological and prospective evidence has established elevated Lp(a) as an independent risk factor for incident cardiovascular disease, with particularly strong associations observed in patients with diabetes [5, 6, 7, 8].

In the setting of secondary prevention, elevated Lp(a) levels have also been shown to independently predict adverse cardiovascular events in patients with established coronary artery disease and in those undergoing PCI [9]. Importantly, substantial residual cardiovascular risk persists despite intensive lipid‐lowering therapy, and recurrent ischemic events continue to occur even among patients achieving low LDL‐cholesterol levels [10]. This has renewed interest in Lp(a) as a contributor to residual risk beyond traditional lipid parameters.

Furthermore, observational data have demonstrated a continuous, dose‐dependent relationship between Lp(a) levels and the risk of recurrent ischemic events following acute coronary syndromes (ACS), suggesting that Lp(a) may serve as a clinically relevant modifier of post‐ACS residual risk [11]. However, existing studies have reported heterogeneous outcomes and have variably focused on composite endpoints, recurrent MI, or repeat revascularization, limiting the ability to draw definitive conclusions in high‐risk subgroups.

To date, no meta‐analysis has systematically quantified the association between elevated [Lp(a)] levels and recurrent MI or repeat coronary revascularization specifically among diabetic patients following ACS or PCI.

The objective of this systematic review and meta‐analysis was to evaluate the association between elevated [Lp(a)] levels and recurrent MI in patients with diabetes and prior ACS or PCI. A secondary objective was to assess the relationship between elevated Lp(a) levels and repeat coronary revascularization in this high‐risk population.

## 2 Materials and Methods

### 2.1 Literature Search

A systematic literature search was conducted in PubMed, EBSCOhost, and the Cochrane Library to identify studies evaluating the association between [Lp(a)] levels and recurrent cardiovascular outcomes in patients with diabetes mellitus following ACS, MI, or PCI.

The search strategy combined Medical Subject Headings (MeSH) terms and keywords as follows: (“lipoprotein(a)” OR “Lp(a)”) AND (“diabetes” OR “type 2 diabetes mellitus”) AND (“myocardial infarction” OR “acute coronary syndrome” OR “percutaneous coronary intervention” OR “PCI”) AND (“recurrent myocardial infarction” OR “revascularization” OR “ischemic events” OR “cardiovascular outcomes”).

The study protocol was prospectively registered in the International Prospective Register of Systematic Reviews (PROSPERO; registration number CRD420261296821). This systematic review and meta‐analysis were conducted and reported in accordance with the Preferred Reporting Items for Systematic Reviews and Meta‐Analyses (PRISMA) guidelines [12].

### 2.2 Studies Selection and Data Extraction

Two independent reviewers (E.E. and M.T.) performed the study selection and data extraction. Discrepancies were resolved through discussion and, when necessary, consultation with a third reviewer (A.P.).

After removal of duplicate records, a two‐stage screening process was applied. First, titles and abstracts were screened for eligibility. Studies were included if they met the following criteria:

- Adult patients with diabetes mellitus.
- History of ACS, MI, or PCI.
- Assessment of Lp(a) levels as the exposure variable.
- Reporting of recurrent MI and/or repeat coronary revascularization as outcomes.
- Provision of hazard ratios (HRs) with 95% confidence intervals (CIs) or sufficient data to derive them.
- Full‐text, peer‐reviewed articles published in English.

Studies were excluded if they:

- Did not include diabetic patients or did not report diabetes‐specific outcomes.
- Did not evaluate Lp(a) as an exposure.
- Reported outcomes unrelated to recurrent ischemic events or revascularization.
- Were case reports, reviews, meta‐analyses, editorials, letters, or conference abstracts.
- Were not published in English.

Full texts of potentially eligible studies were subsequently reviewed, and reasons for exclusion were documented. Data were extracted using a standardized form, including study design, country, sample size, Lp(a) cutoff or categorization, follow‐up duration, outcome definitions, and adjusted effect estimates.

### 2.3 Assessment of Risk of Bias and Quality of Evidence

The methodological quality of included studies was independently assessed by E.E. and M.T. using the Newcastle–Ottawa Scale (NOS) for observational studies. Any disagreements were resolved by consensus with a third reviewer (A.P.).

### 2.4 Data Analysis

The statistical analyses, including the generation of forest plots, were conducted using RevMan software (Review Manager, Version 5.4, The Cochrane Collaboration, London, UK) by three authors (A.P., E.E., and M.T.).

Given that outcomes were time‐to‐event in nature, pooled effect estimates were calculated using HRs with corresponding 95% CIs.

All analyses were conducted using a random‐effects model to account for between‐study heterogeneity. Statistical heterogeneity was assessed using the *I* ^2^ statistic, with values of 25%, 50%, and 75% representing low, moderate, and high heterogeneity, respectively. A two‐sided *p* < 0.05 was considered statistically significant.

Sensitivity analyses were performed by sequential exclusion of individual studies to assess the robustness of pooled estimates. Publication bias was evaluated by visual inspection of funnel plots when applicable.

## 3 Results

In Figure 1, we show the study selection process and results from the literature search. A systematic search was performed in PubMed, EBSCO, and the Cochrane Library up to October 2025. After removal of duplicates and screening of titles and abstracts, 151 full‐text articles were assessed for eligibility. Following full‐text review, A total of seven studies, comprising 7887 participants, were included in the analysis.

**Figure 1:**
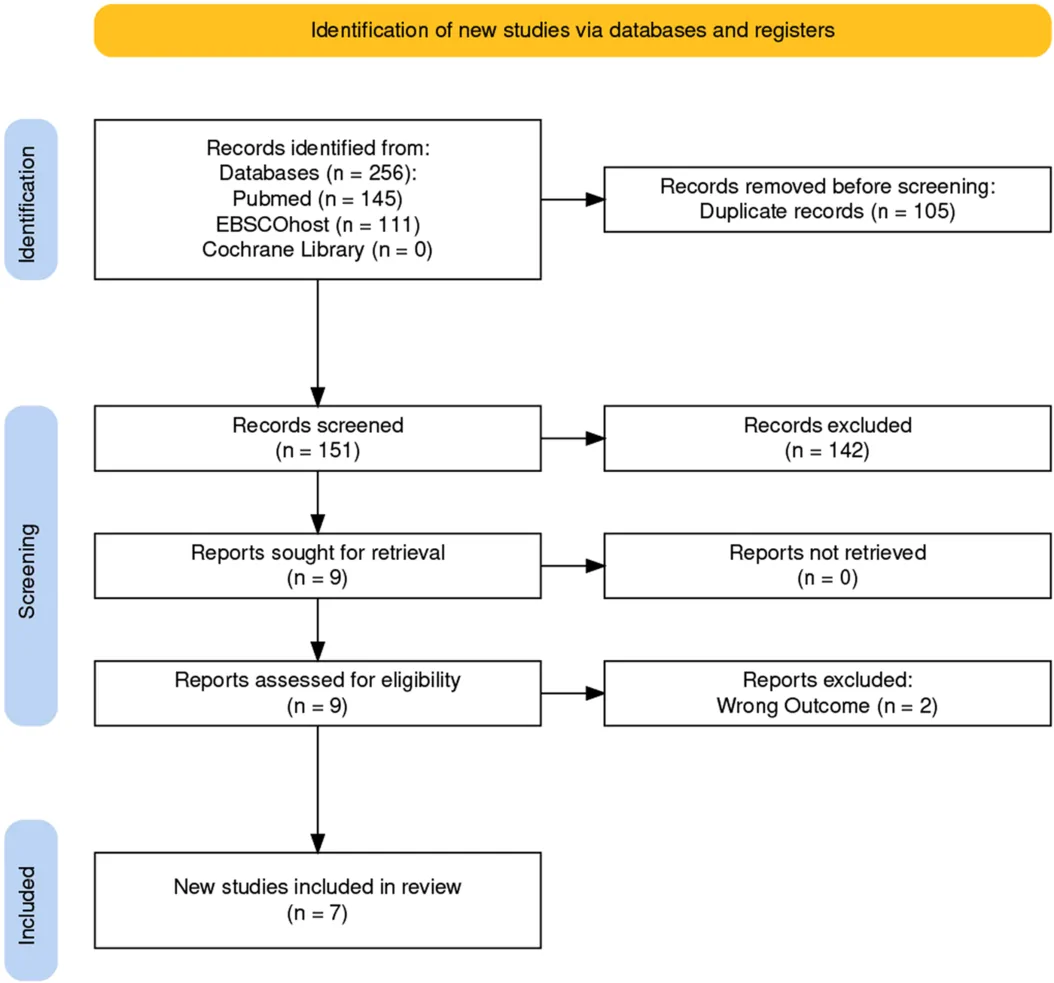
PRISMA 2020 flow diagram of study screening and selection.

### 3.1 Characteristics of Included Studies

Sample sizes ranged from 362 to 2284 individuals. Six studies were prospective cohorts, and one was retrospective. Four studies were conducted in China, two in Italy, and one in South Korea. The mean age of participants ranged from approximately 58 to 66 years. The proportion of individuals with DM2 varied across cohorts; three studies exclusively enrolled patients with DM2, whereas the remaining studies included mixed populations with and without diabetes (Table 1).

**Table 1:** Characteristics of included studies evaluating elevated lipoprotein(a) and recurrent ischemic events in diabetic patients after PCI.

First author	Year	Country	Study design	Population	Overall (*n*)	Diabetes (*n*)	Mean age	Lp(a) cutoff	Upper cutoff *n*	Index event	Primary outcome	Follow‐up
Kwon	2015	Korea	Prospective cohort	T2DM + ACS + PCI	1494	1494	63.5 ± 10.3	Tertiles (T1: 4.7; T2: 13.5; T3: 38.8 mg/dL)	498	MI	Revascularization	4.4 ± 2.6 years
Galasso	2021	Italy	Prospective cohort	STEMI	724	187	62.1 ± 13.3	≥ 30 mg/dL	48	MI	Recurrent MI	Median 803 days (324–1394)
Silverio	2022	Italy	Retrospective cohort	Post‐MI	1018	280	63	< 10 versus ≥ 30 mg/dL	51	MI	Recurrent MI	1121 days
Zhang	2020	China	Prospective cohort	T2DM + MI	2284	2284	58.5	≥ 30 mg/dL	669	MI	Recurrent MI	6 months
Jiang	2025	China	Prospective cohort	T2DM + ACS + PCI	462	462	62.8 ± 11.1	≥ 180 mg/dL	159	MI	Recurrent MI	27 months
Li	2023	China	Prospective cohort	ACS + PCI	1543	678	63.0 (54.0–69.6)	≥ 30 mg/dL	195	MI	Revascularization + Recurrent MI	1446 days
Hao	2022	China	Prospective cohort	T2DM + ACS + PCI	362	362	65.5 ± 9.7	> 21.7 mg/dL	120	MI	Revascularization	2 years

*Note:* Lp(a) concentrations are reported as defined in each individual study. Mean age is presented as mean ± standard deviation or median (interquartile range), as originally reported.

Abbreviations: ACS, acute coronary syndrome; Lp(a), lipoprotein(a); MI, myocardial infarction; PCI, percutaneous coronary intervention; STEMI, ST‐segment elevation myocardial infarction; T2DM, type 2 diabetes mellitus.

All studies included patients with established coronary artery disease and a recent ischemic index event. MI was the index event in all cohorts, including populations with ST‐segment elevation myocardial infarction (STEMI), post‐MI status, ACS treated with PCI, and DM2 with MI or ACS undergoing PCI.

Lp(a) exposure definitions varied across studies. Most cohorts used a threshold of ≥ 30 mg/dL to define elevated Lp(a) levels. One study applied a higher cutoff of ≥ 180 mg/dL, focusing on markedly elevated concentrations. Another categorized Lp(a) into tertiles, while one compared < 10 versus ≥ 30 mg/dL.

Outcome reporting varied across studies, with five providing data for recurrent MI and three for ischemia‐driven revascularization; one study reported a composite endpoint including both outcomes. Follow‐up duration ranged from 6 months to 4.4 years.

### 3.2 Association Between Elevated Lp(a) and Recurrent MI

Five studies were included in the meta‐analysis evaluating the association between elevated Lp(a) levels and recurrent MI in diabetic patients after PCI (Figure 2). After the random‐effects model, elevated Lp(a) was associated with a significantly higher risk of recurrent MI, with a pooled HR of 2.17 (95% CI: 1.02–4.62). Between‐study heterogeneity was substantial (*I* ^2^ = 75%), indicating variability in effect size estimates across cohorts.

**Figure 2:**
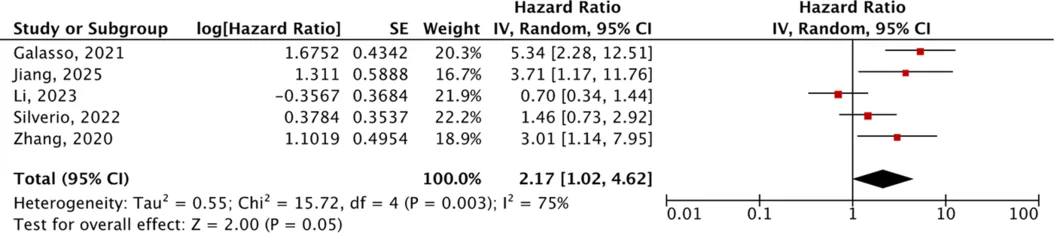
Forest plot of elevated lipoprotein(a) [Lp(a)] and recurrent myocardial infarction in diabetic patients after ACS or PCI.

### 3.3 Association Between Elevated Lp(a) and Ischemia‐Driven Revascularization

Three studies assessed the relationship between elevated Lp(a) and ischemia‐driven revascularization following PCI (Figure 3). The pooled random‐effects estimate showed no statistically significant association between elevated Lp(a) and ischemia‐driven revascularization, with a pooled HR of 1.68 (95% CI: 0.79–3.57). Substantial heterogeneity was observed across studies (*I* ^2^ = 78%), reflecting differences in study design, Lp(a) cut‐offs, and follow‐up duration.

**Figure 3:**
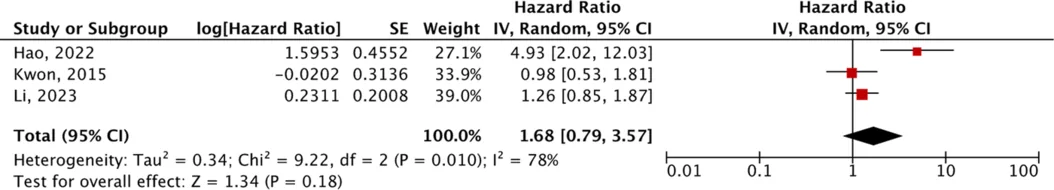
Forest plot of elevated lipoprotein(a) [Lp(a)] and repeat coronary revascularization in diabetic patients after PCI.

## 4 Discussion

In this systematic review and meta‐analysis, including seven studies and 7887 patients with diabetes and prior ACS or PCI, elevated [Lp(a)]levels were associated with a significantly increased risk of recurrent MI. The pooled analysis demonstrated that patients with elevated Lp(a) had more than a twofold higher risk of recurrent MI compared with those with lower levels. In contrast, elevated Lp(a) was not significantly associated with ischemia‐driven repeat coronary revascularization. Although substantial heterogeneity was observed across studies, the overall direction of the association between elevated Lp(a) and recurrent MI remained consistent. These findings suggest that Lp(a) may represent an important contributor to residual ischemic risk among diabetic patients following ACS or PCI.

The substantial between‐study heterogeneity observed for recurrent MI (*I* ^2^ = 75%) likely reflects several clinically and methodologically important differences among the included cohorts. Diabetes ascertainment was not uniform. Some studies defined diabetes primarily according to a documented history and/or current glucose‐lowering treatment, whereas others incorporated biochemical criteria such as HbA1c ≥ 6.5%, fasting plasma glucose ≥ 7.0 mmol/L (126 mg/dL), or a 2 h oral glucose tolerance test value ≥ 11.1 mmol/L. Lp(a) exposure definitions also differed considerably. Studies used thresholds ranging from 10 to 70 mg/dL (with commonly applied cutoffs of 30 and 50 mg/dL), while others used substantially higher thresholds, including ≥ 180 mg/dL. Clinical settings were also heterogeneous, including STEMI treated with primary or emergency PCI, broader post‐MI populations, ACS with T2DM undergoing PCI, and stable CAD with previous cardiovascular events. These differences in baseline risk, Lp(a) exposure contrasts, diabetes ascertainment, and clinical presentation may have contributed to the variability in effect estimates for recurrent MI.

[Lp(a)] is a low‐density lipoprotein–like particle composed of apolipoprotein B100 covalently bound to apolipoprotein(a), a highly polymorphic glycoprotein [13]. Elevated Lp(a) levels exert both proatherogenic and prothrombotic effects that contribute to the development and progression of cardiovascular disease. Lp(a) carries oxidized phospholipids that promote vascular inflammation and accelerate atherosclerotic plaque progression. In addition, the structural similarity between apolipoprotein(a) and plasminogen interferes with fibrinolysis, favoring thrombosis and platelet activation [14, 15].

The interaction between Lp(a) and T2DM may further amplify these mechanisms. Although some studies have reported an inverse relationship between Lp(a) levels and insulin resistance, individuals with T2DM remain at markedly increased cardiovascular risk [16]. Diabetes is characterized by endothelial dysfunction, oxidative stress, and chronic inflammation, all of which accelerate atherosclerosis and plaque instability. Moreover, Lp(a) has been associated with increased carotid intima‐media thickness in patients with T2DM, suggesting a role in the progression of vascular disease in this population [1, 17, 18, 19]. Similarly, studies have shown that Lp(a) is associated with an increased risk of both micro and macrovascular complications in diabetes, leading to enhanced damage to the vascular endothelium due to a synergistic effect between Lp(a) and glucose [20]. Together, these mechanisms may explain why elevated Lp(a) levels contribute to the heightened risk of recurrent ischemic events observed in diabetic patients.

Patients who experience ACS remain at substantial risk for recurrent major adverse cardiovascular events (MACE), with event rates approaching 33.4% at 5 years of follow‐up [21]. Contemporary secondary prevention strategies have largely focused on aggressive reduction of low‐density lipoprotein cholesterol (LDL‐C). However, even with intensive LDL‐C lowering, a considerable residual risk of cardiovascular events persists, suggesting the involvement of additional pathogenic factors [22]. Several cohort studies in patients with coronary artery disease have demonstrated that elevated Lp(a) levels are associated with worse clinical outcomes after PCI. Mojahedi et al. reported that higher Lp(a) concentrations are linked to premature and more severe presentations of ACS and are associated with a greater risk of recurrent ischemic events both early after the index event and during long‐term follow‐up [23]. Similarly, Willeit et al., analyzing 29 069 participants from seven placebo‐controlled statin trials, showed that elevated Lp(a) independently predicted cardiovascular events in statin‐treated patients. Importantly, as LDL‐C–related risk was reduced, Lp(a)‐associated risk became a more prominent contributor to residual cardiovascular risk, particularly at levels exceeding 50 mg/dL [24].

In the ACS setting, additional studies have also demonstrated a relationship between Lp(a) and recurrent ischemic outcomes. Miñana et al. reported that elevated Lp(a) levels measured during hospitalization for acute MI were associated with an increased risk of long‐term recurrent reinfarction [25]. Likewise, Yang et al. found that higher Lp(a) concentrations independently predicted recurrent cardiovascular events and unplanned revascularization among patients with ACS [26].

The present meta‐analysis extends these findings by specifically evaluating the prognostic impact of elevated Lp(a) in patients with diabetes following ACS or PCI. Our findings support Lp(a) as an independent predictor of recurrent MI and suggest that its measurement may improve post‐ACS risk stratification, particularly in high‐risk patients with diabetes.

Despite major advances in secondary prevention—including high‐intensity statins, dual antiplatelet therapy, and the use of new‐generation DES—patients with coronary artery disease continue to experience recurrent ischemic events. This persistent burden of events highlights the importance of residual cardiovascular risk factors beyond LDL‐C. [Lp(a)] has emerged as one of the most important non‐LDL contributors to this residual risk. Lp(a) concentrations are largely genetically determined, with heritability estimates ranging from 70% to 90%, and are minimally influenced by conventional lipid‐lowering therapies. Notably, statins do not reduce Lp(a) levels and may even increase them by approximately 10%–20% through upregulation of the LPA gene. Nevertheless, this effect does not diminish the well‐established cardiovascular benefits of statin therapy and does not support their discontinuation [27, 28].

Observational evidence supports the prognostic role of Lp(a) in high‐risk populations. Konishi et al. reported that elevated Lp(a) levels were associated with increased cardiac death and ACS after PCI in patients with type 2 diabetes [29], while Li et al. showed that higher Lp(a) concentrations were associated with a greater risk of MACE [20]. Similarly, Galasso et al. reported that elevated Lp(a) levels, particularly at concentrations ≥ 30 mg/dL, were associated with recurrent MI in patients with STEMI [30]. In contrast, repeat revascularization may be influenced by factors beyond systemic atherothrombotic risk. Although new‐generation DES have substantially reduced neointimal hyperplasia, in‐stent restenosis remains an important mechanism of PCI failure, with procedural and anatomical factors such as small vessel size, long stented segments, residual stenosis, and stent drug type contributing to its development [31]. These factors may partly explain why elevated Lp(a) was not significantly associated with repeat revascularization in our analysis.

[Lp(a)] levels are largely genetically determined and remain relatively stable throughout life, with minimal influence from age, sex, lifestyle factors, diabetes status, or most medications. Statins, the cornerstone of ASCVD prevention, have little effect on Lp(a) concentrations, and other lipid‐lowering therapies produce only modest reductions [8, 32]. Given its stability and ease of measurement, Lp(a) may serve as a useful biomarker for risk stratification in patients with T2DM undergoing PCI.

Emerging therapies targeting Lp(a) are currently under investigation. PCSK9 inhibitors produce modest reductions in Lp(a), while novel RNA‐based therapies such as olpasiran, muvalaplin, and pelacarsen have demonstrated reductions of up to 80% in clinical trials [22, 33]. Further studies are needed to determine the degree of Lp(a) lowering required to achieve meaningful cardiovascular benefit.

This study has several strengths. To our knowledge, this is the first meta‐analysis specifically evaluating the association between elevated [Lp(a)] levels and recurrent MI in patients with diabetes following ACS or PCI. By pooling data from multiple cohort studies, our analysis provides a more comprehensive estimate of the prognostic impact of Lp(a) in this high‐risk population. Additionally, the included studies reported adjusted HRs and evaluated time‐to‐event outcomes, allowing for a more reliable assessment of the relationship between Lp(a) and recurrent ischemic events.

### 4.1 Limitations

Several limitations should also be acknowledged. First, all included studies were observational in design, which introduces the possibility of residual confounding despite multivariable adjustment. Second, substantial heterogeneity was observed across studies, likely reflecting differences in Lp(a) cutoffs, patient populations, and follow‐up durations. Third, the number of studies evaluating repeat revascularization was limited, which may have reduced statistical power to detect a significant association. Finally, most studies were conducted in Asian populations, which may limit the generalizability of the findings to other populations.

## 5 Conclusion

In summary, this study demonstrates that elevated [Lp(a)] levels are associated with a significantly increased risk of recurrent MI in patients with diabetes following ACS or PCI. These findings highlight the potential role of Lp(a) as an important contributor to residual cardiovascular risk in this high‐risk population. Incorporating Lp(a) assessment into post‐ACS risk stratification may help identify patients who could benefit from closer monitoring and emerging Lp(a)‐targeted therapies.

## Author Contributions

**Ermilo Echeverría‐Ortegón:** conceptualization, methodology, formal analysis, data curation, visualization, writing–original draft, writing – review and editing. **Mario Torres:** methodology, data curation, validation, writing – review and editing. **Alfredo Pérez‐Tagle Tejeda:** conceptualization, supervision, writing – review and editing, project administration. **Paulina Caballero‐Osorno:** data curation, investigation, writing – review and editing. **Juan Pastor‐Cervantes:** conceptualization, supervision, writing – review and editing, clinical expertise. **Hugo Laviada‐Molina:** conceptualization, supervision, writing–review and editing, clinical expertise.

## Funding

The authors have nothing to report.

## Ethics Statement

Ethical approval was not required for this study because it is a systematic review and meta‐analysis based exclusively on previously published data.

## Consent

Patient consent was not required because no individual patient data were collected or analyzed in this study.

## Conflicts of Interest

The authors declare no conflicts of interest.

## Data Availability

The data supporting the findings of this study are derived from previously published studies and are available in the cited references. Extracted data sets used in the analysis are available from the corresponding author upon reasonable request.
